# Cigarette smoking induces human CCR6^+^Th17 lymphocytes senescence and VEGF-A secretion

**DOI:** 10.1038/s41598-020-63613-4

**Published:** 2020-04-16

**Authors:** Indoumady Baskara, Stéphane Kerbrat, Maylis Dagouassat, Hoang Quy Nguyen, Maude Guillot-Delost, Mathieu Surenaud, Claude Baillou, François M. Lemoine, Didier Morin, Jorge Boczkowski, Sabine Le Gouvello

**Affiliations:** 1 Université Paris-Est, Créteil, 94000 France; 2 Inserm, UMR 955, équipe 4, Créteil, F-94010 France; 3 Inserm, UMR 955, équipe 7, Créteil, F-94010 France; 4 Institut Curie, PSL Research University, Paris, France; 5 Inserm, UMR 932, F-75005 Paris, France; 6 Inserm, UMR 955, équipe 16, Créteil, 94000 France; 7 Sorbonne Université, UPMC Univ-Paris 06, CIMI-Paris- INSERM UMR U 1135, Paris, France; 8 Inserm, UMR 955, équipe 3, Créteil, F-94010 France; 9 AP-HP, Hôpital H. Mondor- A. Chenevier, Pôle de Biologie-Pathologie, Créteil, 94000 France

**Keywords:** Senescence, T-helper 17 cells

## Abstract

Chronic exposure to environmental pollutants is often associated with systemic inflammation. As such, cigarette smoking contributes to inflammation and lung diseases by inducing senescence of pulmonary cells such as pneumocytes, fibroblasts, and endothelial cells. Yet, how smoking worsens evolution of chronic inflammatory disorders associated with Th17 lymphocytes, such as rheumatoid arthritis, psoriasis, Crohn’s disease, and multiple sclerosis, is largely unknown. Results from human studies show an increase in inflammatory CD4^+^ Th17 lymphocytes at blood- and pulmonary level in smokers. The aim of the study was to evaluate the sensitivity of CD4^+^ Th17 lymphocytes to cigarette smoke-induced senescence. Mucosa-homing CCR6^+^ Th17- were compared to CCR6^neg^ -and regulatory T peripheral lymphocytes after exposure to cigarette smoke extract (CSE). Senescence sensitivity of CSE-exposed cells was assessed by determination of various senescence biomarkers (β-galactosidase activity, p16^Ink4a^- and p21 expression) and cytokines production. CCR6^+^ Th17 cells showed a higher sensitivity to CSE-induced senescence compared to controls, which is associated to oxidative stress and higher VEGFα secretion. Pharmacological targeting of ROS- and ERK1/2 signalling pathways prevented CSE-induced senescence of CCR6^+^Th17 lymphocytes as well as VEGFα secretion. Altogether, these results identify mechanisms by which pro-oxidant environmental pollutants contribute to pro-angiogenic and pathogenic CCR6^+^Th17 cells, therefore potential targets for therapeutic purposes.

## Introduction

Substantial epidemiologic evidence links environmental pollutants to adverse health effects through an inflammatory pathway. As such, cigarette smoking plays dual role in regulating immunity by either attenuation of defensive immunity or exacerbation of pathogenic immune responses^1^. Prevalence of smoking is higher in patients with inflammatory diseases such as chronic obstructive pulmonary disease (COPD) and various pathogenic Th17-associated autoimmune disorders such as rheumatoid arthritis, psoriasis, Crohn’s disease, and multiple sclerosis; moreover, smoking worsens the evolution of these diseases^2^. While the mechanism of cigarette smoke affecting the lung is well known, its effect on CD4^+^ T helper (Th) lymphocytes is still incompletely understood.

CD4+ Th lymphocytes differentiate upon combined activation through the T cell receptor for antigen (TCR), costimulatory signals through CD28 receptor and appropriate polarizing cytokines into different subsets (e.g. Tfh, Th1, Th2, Th17, regulatory T cell). Those are characterized mainly by their distinct master transcription factors as well as the cytokines they produce and ultimately their functions against invading pathogens or in mediating tolerance^3^. IL-17-producing Th17 cells protect against extracellular bacterial and fungi infections by secreting cytokines IL-17A, IL-17F and IL-22. However, so-called “pathogenic” Th17 have been described as contributing to the pathogenesis of several human chronic inflammatory diseases via excessive and/or aberrant cytokine production^3^. The chemokine receptor CCR6 (CD196) was identified as the main surface marker characterizing the Th17 lineage, and regulating the recruitment of Th17 cells into inflamed tissues under physiological as well as inflammatory conditions^3^. Data from human studies show an increase in CD4^+^ Th17 cells absolute number and proportion, in the blood and lungs of smokers^4–7^. Accordingly, cigarette smoke-induced pulmonary inflammation is attenuated in CCR6-deficient mice by preventing mucosa-homing of CCR6^+^Th17 cells^8^. However, the direct influence of cigarette smoke exposure towards CCR6^+^Th17 cells and its pathogenic consequences, remain largely unknown.

Various studies show an increased number of senescent cells in lungs and peripheral leucocytes from smokers with normal lung function and even higher in patients with COPD^9–11^. Cellular senescence is recognized as a cell-cycle arrest and is induced by replicative proliferation or various stresses, including oxidative stress^12^. Replicative senescence as well as stress-induced senescence show similar phenotypic features such as increased activity of senescence-associated-β-galactosidase (SA-β-Gal), relative resistance to apoptosis, and activation of the ATM/ATR-p53-p21^Cdkn1a^ and/or the p16^INK4a^ -retinoblastoma protein (pRb) pathways. Senescent cells usually secrete a complex mix of mostly pro-inflammatory factors termed as senescence-associated secretory phenotype (SASP), which can have detrimental effects on organs function^12^.

In the present study, we hypothesized that cigarette smoke could induce senescence of resting Th17 cells, which in turn will promote pathogenic secretory protein profiles in smokers, independently of any infectious challenge. In order to examine this hypothesis, we first analyzed the sensitivity of non-reactivated memory Th17 cells to senescence. To do so, we used an *in vitro* standardized method^13^ by testing acute effects of cigarette smoke extract (CSE) on CD4^+^ CD45RO^+^ CCR6^+^ Th17 memory cells from healthy donors. Secondly, we examined the molecular mechanisms involved during this process, by focusing on oxidants and ERK1/2 pathway.

## Results

### CCR6^+^ Th17 cells are highly susceptible to cigarette smoke-induced senescence

**Figure 1:**
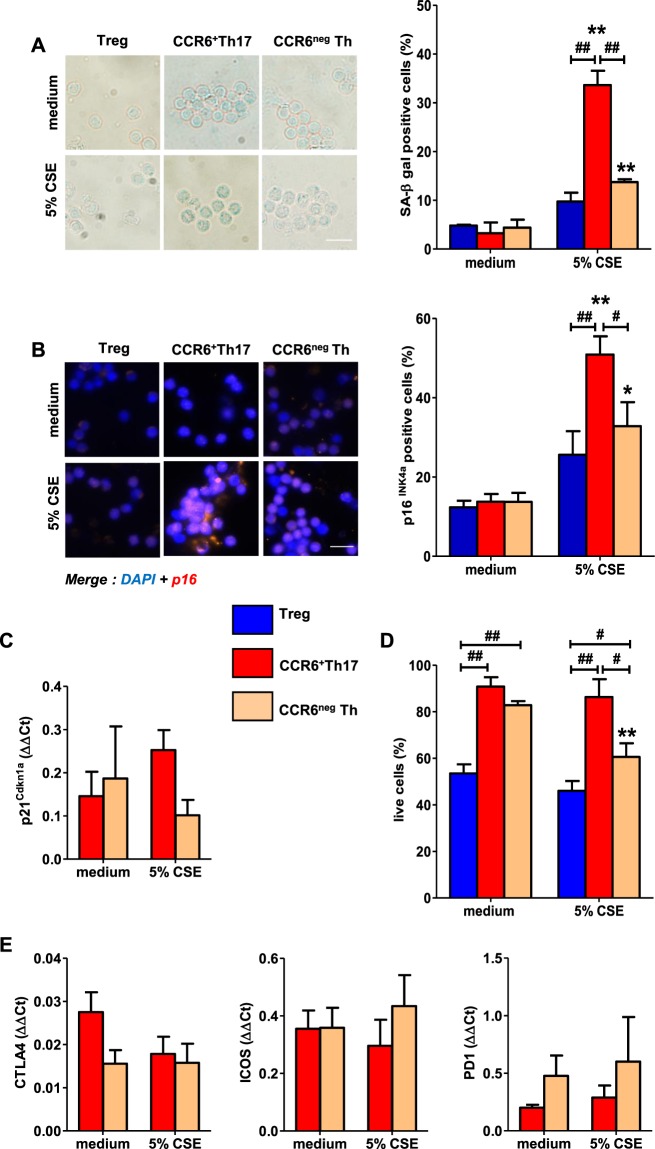
Exposure to cigarette smoke induces premature senescence of human CCR6^+^Th17 cells. CD4^+^T cell subpopulations CCR6^+^Th17, CCR6^neg^Th and Treg cells were exposed to 5% cigarette smoke extract (CSE). Various senescence hallmarks were analyzed at indicated times, relevant to senescence implementation timing. Representative images for the indicated conditions and quantitation of (**A**) SA β-gal positive cells exhibiting cytoplasmic blue dot staining at 48 h (n = 3.); (scale bar = 20 µm). (**B**) p16^INK4a^ positive cells exhibiting nuclear red dot staining at 24 h (n = 8); nuclei are stained with DAPI (scale bar = 20 µm). Data are presented as means ± SEM. (**C**,**E**) Gene expression analysis by qRT-PCR of p21^Cdkn1a^ at 3 h, and exhaustion markers ICOS, CTLA4, PD1 at 48 h (n = 5). (**D**) Cell viability analyzed at 48 h after 5% CSE exposure, by enumeration of cells excluding trypan blue (n = 8). Statistical analysis by Mann-Whitney test; ^#^p < 0.05, ^##^p < 0.01: comparison between 2 sub-populations; *p < 0.05, **p < 0.01: comparison to medium condition.

**Figure 2:**
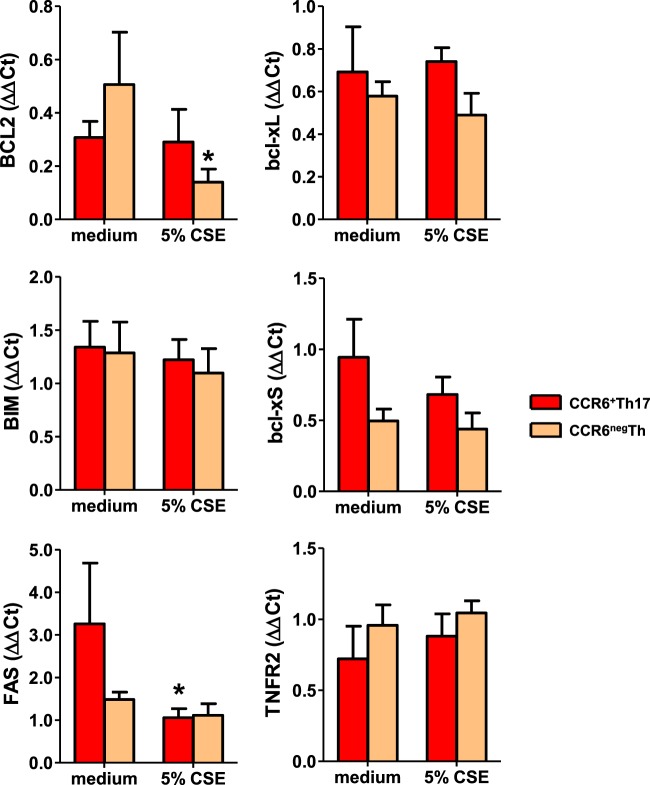
Cigarette smoke induced- senescent CCR6^+^Th17 cells have decreased pro-apoptotic sensitivity. Gene expression analysis performed by qRT-PCR for Bcl-xL, Bcl-xS, BIM, FAS, TNFR2 (1h30 after 5% cigarette smoke extract (CSE) exposure) and Bcl2 (6 h after 5% CSE exposure) (n = 5). Statistical analysis by Mann-Whitney test; ^#^p < 0.05, ^##^p < 0.01: comparison between 2 sub-populations; *p < 0.05, **p < 0.01: comparison to medium condition.

To analyze the senescence susceptibility of Th17 cells to CSE, we evaluated three hallmarks of senescence: SA β-gal activity, p16^INK4a^ and p21^Cdkn1a^ expression^14^. Resting CCR6^+^ CD45RO+ Th17 memory cells (henceforth referred to as “CCR6^+^Th17”) were treated with non-toxic doses of CSE (Fig. S1A), and compared to CSE-exposed resting CCR6^neg^ T effector/memory lymphocytes (henceforth referred to as “CCR6^neg^Th”) and regulatory T cells (Treg). CSE exposure induced SA β-gal activity both in CCR6^+^Th17 and CCR6^neg^Th cells, but not in Treg. The proportion of SA β-gal positive cells was significantly more important among CCR6^+^Th17 compared to CCR6^neg^Th cells (Fig. 1A). Similar effects were observed for the expression of p16^INK4a^ in CCR6^+^Th17 and CCR6^neg^Th cells, after 5% CSE treatment (Fig. 1B). Conversely, p21^Cdkn1a^ expression in CCR6^+^Th17 and CCR6^neg^Th cells was not significantly different compared to controls, after CSE exposure (Fig. 1C). The proportion of live CCR6^+^Th17 cells, observed after 48 hours treatment with CSE 5%, attested their absence of proliferation (Figs. 1D & S1A). In addition, the level of transcripts such as CTLA4, ICOS and PD-1, associated with replicative senescence-induced exhaustion^15^ was unchanged after CSE exposure (Fig. 1E). As resistance to apoptosis is one of the hallmarks of senescence^14^, we analyzed the expression of pro- and anti-apoptotic effectors (Bcl2, Bcl-xL, Bcl-xS, Bim, Fas, TNFR2) after exposing cells to CSE. Only CCR6^+^Th17 cells showed a decrease in the pro-apoptotic gene Fas expression, and only CCR6^neg^Th cells presented a decrease in the anti-apoptotic Bcl2 gene expression compared to controls, after treatment of cells with CSE (Fig. 2); no modulation of the other genes expression was observed after CSE exposure.

**Figure 3:**
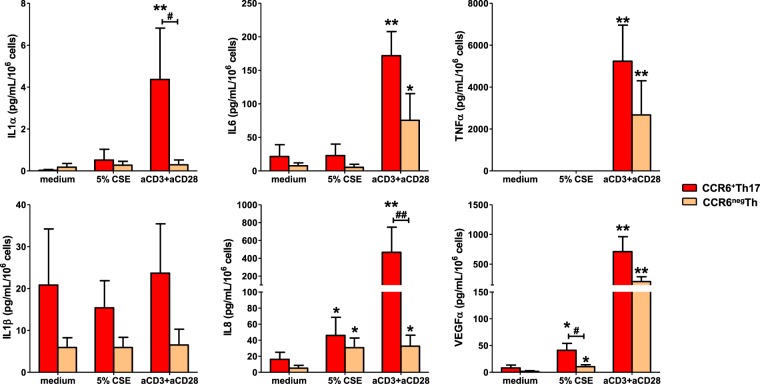
Cigarette smoke induced- senescent CCR6^+^Th17 cells secrete VEGFα independently of TCR-dependent reactivation. SASP components analysis by Luminex assay, in cell culture supernatants, at 48 h after 5% cigarette smoke extract (CSE) exposure (n = 8). Plate-bound anti-CD3ε antibody (5 µg/mL) and soluble anti-CD28 antibody (2 µg/mL) simultaneous stimulation used as positive control. Statistical analysis by Mann-Whitney test; ^#^p < 0.05, ^##^p < 0.01: comparison between 2 sub-populations; *p < 0.05, **p < 0.01: comparison to medium condition.

We then quantified the secretion of usual SASP components^12^ in culture supernatants of CSE-exposed- CCR6^+^Th17 and CCR6^neg^Th cells **(**Fig. S1B**)**, as we evaluated the expression profile of those cells for IL-17, IL-22, IL-21, CCL20 (Th17 signature cytokines), IFN-γ (Th1 signature cytokine), IL-4, IL-5, IL-13 (Th2 signature cytokines), IL-10 and TGFβ (expressed by Treg)^1^. Stimulation with anti-CD3 and anti-CD28 mAbs, mimicking antigen-dependent activation, was used as positive control. Compared to anti-CD3/CD28-dependent activation, CSE-exposure of CCR6^+^Th17 and CCR6^neg^Th cells resulted in a similar secretion of IL-1β, a moderate secretion of IL-8 and VEGFα, and no secretion of the other SASP factors, including Il-1α, IL-6 and TNFα (Fig. 3). Interestingly, CSE-exposed resting CCR6^+^Th17 cells secreted larger amounts of VEGFα compared to CSE-exposed CCR6^neg^Th cells. In addition, Th signature cytokines secretion was not induced by CSE exposure and basal CCL20 secretion was inhibited in Th17 cells, although anti-CD3/CD28-dependent activation induced the secretion of most of them (Fig. S1C).

### ERK1/2 pathway governs high susceptibility of CCR6^+^ Th17 cells to CSE-induced senescence

**Figure 4:**
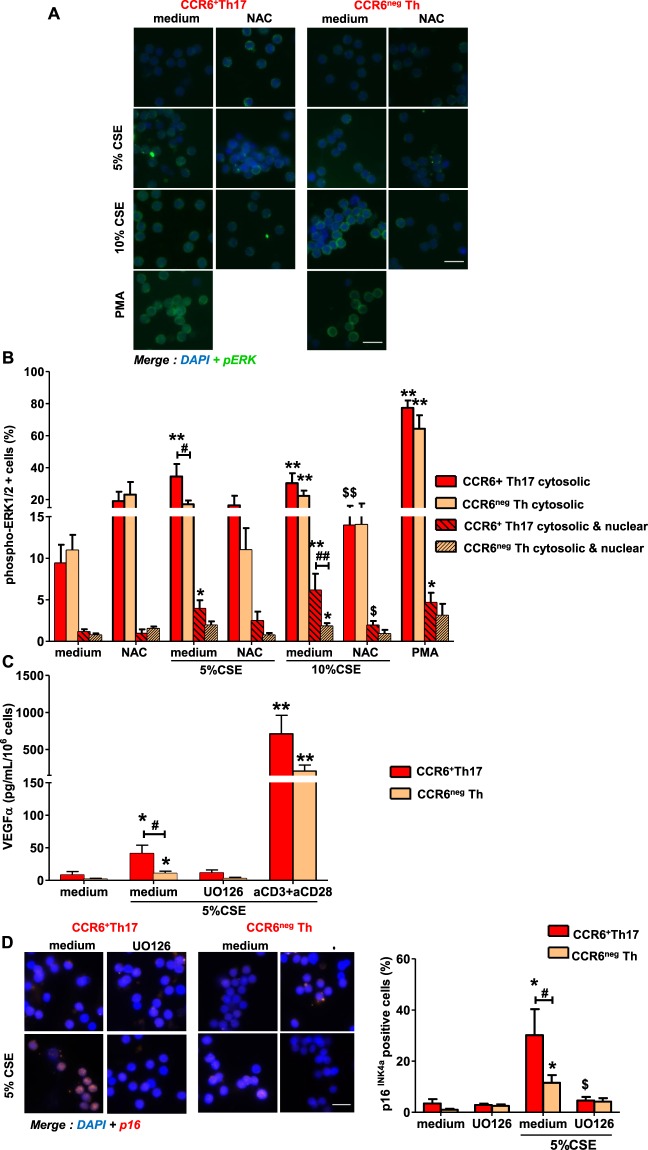
MEK-ERK1/2 activation is required for cigarette smoke-driven p16^INK4a^ expression and VEGFα secretion in CCR6^+^Th17 cells. Cells were exposed to 5% cigarette smoke extract (CSE) or 10% CSE or 200 nM PMA for 15 minutes after (or not) 1h30 pretreatment with 10 mM NAC. (**A**) ERK1/2 activation and localization were assessed by immunofluorescence double staining with anti-phospho-ERK1/2 antibody and DAPI (scale bar = 20 µm). (**B**) Histograms represent the percentage of enumerated cells presenting cytosolic phospho-ERK1/2 staining or cytosolic and nuclear phospho-ERK1/2 staining (n = 10). Data shown are means ± SEM. (**C,D**) Effects of 30-minutes pretreatment with a MEK-ERK1/2 inhibitor UO126 (5 µM) on VEGFα secretion (**C**; n = 4) and p16^INK4a^ expression (**D**; n = 8). Cell treatment and procedure are identical to those described in Figs. 1B & 3. Statistical analysis by Mann-Whitney test; ^#^p < 0.05, ^##^p < 0.01: comparison between 2 sub-populations; *p < 0.05, **p < 0.01: comparison to medium condition; ^$^p < 0.05: comparison to “medium + CSE condition”.

ERK1/2 pathway has been implicated in tumor-induced and regulatory T cell-induced senescence of T cells^16,17^. Moreover, we previously shown that constitutive ERK1/2 tonic activity was more intense in CCR6^+^Th17 cells than in CCR6^neg^Th cells^18^. Therefore, we investigated the potential role of ERK1/2 pathway in CSE-induced CCR6^+^Th17 cells senescence. As shown on Fig. 4A,B, significant nuclear staining of phosphorylated form of ERK1/2 (phospho-ERK1/2) was observed in CCR6^+^Th17 cells after 5% CSE exposure; this effect was significantly more pronounced compared to controls, after 10% CSE exposure. Moreover, 5% CSE treatment significantly increased the number of CCR6^+^Th17 cells presenting cytosolic phospho-ERK1/2 staining, compared to CCR6^neg^Th cells. This difference is lost after 10% CSE exposure. Likewise, PMA stimulation induced significant higher nuclear staining of phospho-ERK1/2 in CCR6^+^Th17 cells compared to controls, although cytosolic phospho-ERK1/2 staining was observed at similar level in both cell types (Fig. 4A,B). Finally, pretreatment with MEK/ERK1/2 inhibitor, UO126, prevented secretion of VEGFα (Fig. 4C) and expression of p16^INK4a^ (Fig. 4D) induced by CSE treatment of both subtypes.

Reactive oxygen species (ROS) modulate ERK1/2 activity and its regulation is required for T cell survival, proliferation and cytokine production^19^. To investigate the direct involvement of ROS in CSE-induced senescence of CCR6^+^Th17 cells, we examined the effect of pan free-radical scavenger N-acetyl-cysteine (NAC) on CSE-dependent ERK1/2 activation. Pretreatment with NAC prevented nuclear staining of phospho-ERK1/2 induced in CCR6^+^Th17 cells by CSE exposure. Similarly, CCR6^+^Th17, pretreated with NAC, presented similar level of cytosolic phospho-ERK1/2 staining compared to control cells, regardless CSE doses used **(**Fig. 4B**)**.

### High sensitivity of CCR6+ Th17 cells to cigarette smoke–induced senescence is associated with higher ROS production

**Figure 5:**
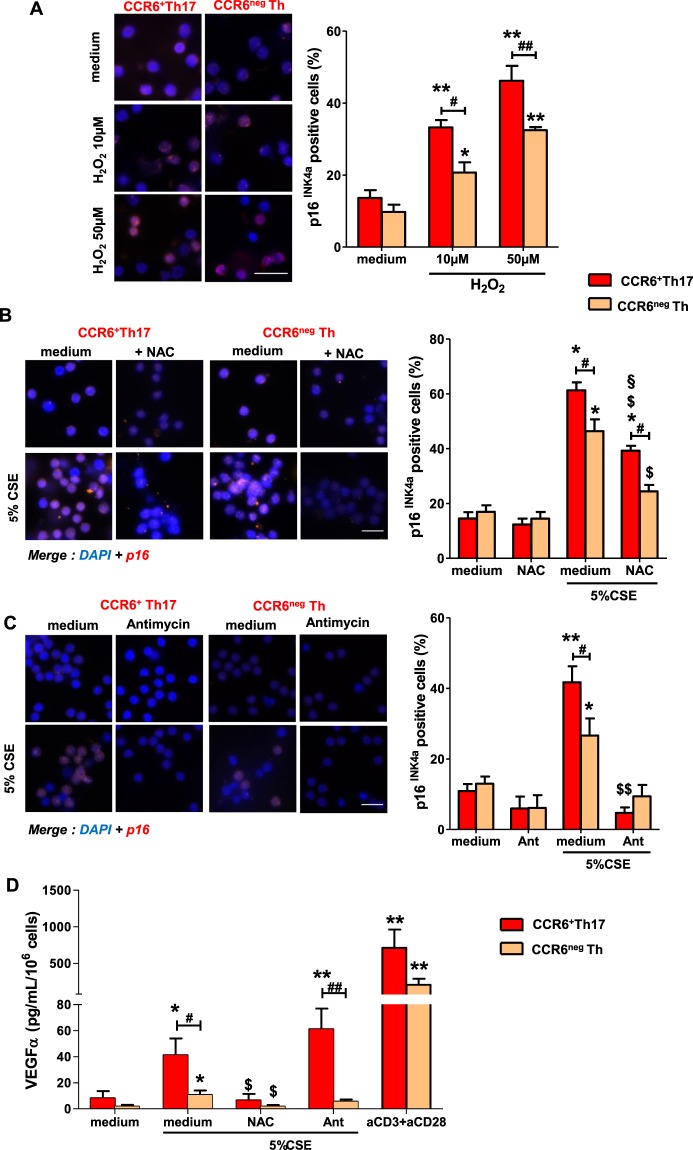
Different ROS sources control cigarette smoke-driven p16^INK4a^ expression and VEGFα secretion in CCR6^+^Th17 cells. (**A**) Representative images and quantitation of p16^INK4a^ positive cells among CCR6^+^Th17 and CCR6^neg^Th cells treated by H_2_O_2_ for 72 h (scale bar = 20 µm; n = 5). Cells were pretreated with 10 mM NAC for 1h30 (**B,D**), or 0,5 µM Ant for 30 minutes (**C,D**), prior to 5% cigarette smoke extract (CSE) exposure. Quantitation of p16^INK4a^ positive cells were performed at 24 h among cells pretreated with (**B**) NAC (n = 6), or (**C**) Ant (n = 4), (scale bar = 20 µm). (**D**) NAC and Ant pretreatment effect on VEGFα secretion (n = 8). Cell treatment and procedure are identical to those described in Fig. 3. Statistical analysis by Mann-Whitney test; ^#^p < 0.05, ^##^p < 0.01: comparison between 2 sub-populations; *p < 0.05, **p < 0.01: comparison to medium condition; $ p < 0.05: comparison to “medium + CSE condition”; ^§^p < 0.05: comparison to corresponding condition w/o CSE.

**Figure 6:**
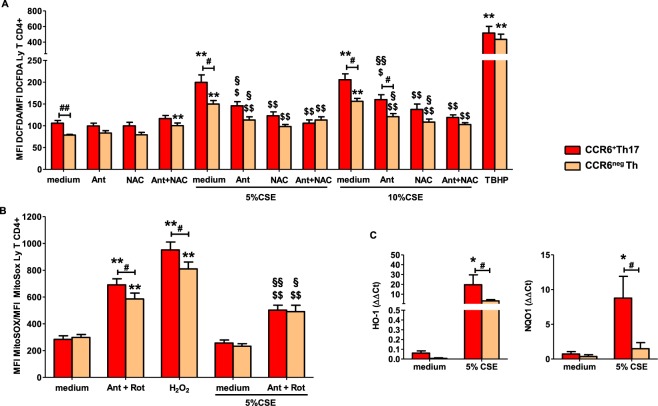
Higher mitochondrial activity is critical for cigarette smoke-driven p16^INK4a^ expression in CCR6^+^Th17 cells. (**A**) Cells were exposed (or not) to 5% cigarette smoke extract (CSE) or 10% CSE or 250 µM TBHP (positive control) for 30 minutes after (or not) pretreatment with 10 mM NAC for 1h30 or 0,5 µM Ant for 30 minutes, or the combination of Ant and NAC. ROS production was measured by flow cytometry using H_2_DCF-DA probe oxidation (n = 5). (**B**) Flow cytometry analysis of MitoSOX red probe oxidation in CCR6^+^Th17 and CCR6^neg^Th cells, pretreated by a combination of 0,5 µM Ant and 2 µM Rotenone for 30 minutes, exposed (or not) to 5% CSE or 125 µM H_2_O_2_ (positive control) for 3 h (n = 5). Data shown (mean ± SEM) are expressed in mean fluorescence intensity (MFI) of the probe in the different cell types and conditions, normalized to the MFI in total CD4^+^ T cells in medium condition. (**C**) HO-1 and NQO1 gene expression was analyzed by qRT-PCR in 5% CSE –exposed cells for 6 h (n = 5). Statistical analysis by Mann-Whitney test; ^#^p < 0.05, ^##^p < 0.01: comparison between 2 sub-populations; *p < 0.05, **p < 0.01: comparison to medium condition; ^$^p < 0.05, ^$$^p < 0.01: comparison to “medium + CSE condition”; ^§^p < 0.05, ^§§^p < 0.01: comparison to corresponding condition w/o CSE.

CSE contains more than 4500 chemicals, in which some of them are well-known sources of exogenous ROS^20^. Moreover, endogenous cellular production of ROS is increased in response to CSE exposure, in smokers^21^. To further investigate the involvement of ROS in our system of interest, we first treated CCR6^+^Th17 and CCR6^neg^Th cells with exogenous H_2_O_2_. p16^INK4a^ expression was increased in H_2_O_2_-treated CD4+ T cells compared to non-treated cells suggesting the contribution of ROS in CSE-induced senescence of CCR6^+^Th17 and CCR6^neg^Th cells. A greater sensitivity of CCR6^+^Th17 cells to ROS-associated senescence is also observed in comparison with CCR6^neg^Th cells **(**Fig. 5A**)**. Then, we tested the effect of NAC pretreatment on CCR6^+^Th17 cells and the induction of p16^INK4a^ expression and VEGFα production after CSE exposure. Since mitochondrial metabolism is a critical component of T cell activation^22^, we also tested the effects of pretreatment with rotenone and/or antimycin A (Ant), two inhibitors of mitochondrial complex I and complex III respectively. NAC pretreatment mitigated CSE- induced increase in p16^INK4a^ expression in both CCR6^+^Th17 and CCR6^neg^Th cells. However, in NAC-pretreated cells, CSE exposure induced a higher proportion of p16^INK4a^-positive CCR6^+^Th17 cells compared to controls (Fig. 5B). Pretreatment with Ant prevented this effect on p16^INK4a^ expression in CCR6^+^Th17 and CCR6^neg^Th cells (Fig. 5C**)**. Simultaneous pretreatment with rotenone and Ant induced similar results *(data not shown)*. Pretreatment with NAC inhibited VEGFα production induced by CSE treatment in both cell subtypes, whereas Ant pretreatment had no effect (Fig. 5D). We further explored the ROS involvement in CSE-induced senescence of Th cells, by testing the effect of CSE exposure on H_2_DCF-DA probe oxidation, which is mostly dependent on the presence of H_2_O_2_^23^. Basal H_2_DCF-specific fluorescence was higher in resting CCR6^+^Th17 compared to resting CCR6^neg^Th cells (Fig. 6A). This difference of basal fluorescence intensity between the two resting cellular subpopulations was deleted by performing a pretreatment with either Ant or NAC or both (Fig. 6A). In response to CSE exposure, H_2_DCF -specific fluorescence intensity was increased in both sub-populations and the intensity enhancement was similar in CCR6^+^Th17 and in CCR6^neg^Th cells (1.5 fold). Independent pretreatment of cells with either Ant or NAC or both reduced H_2_DCF-specific fluorescence in CSE-exposed CCR6^+^Th17 and CCR6^neg^Th cells. These pretreatments were also associated with an abolition of the difference in H_2_DCF-specific fluorescence observed between 5% CSE-exposed CCR6^+^Th17 and CCR6^neg^Th cells; however, H_2_DCF-specific fluorescence remained higher in CCR6^+^Th17 cells pretreated with Ant and after 10% CSE exposure, compared to controls **(**Fig. 6A**)**. As mitochondrial-derived superoxide (O_2_^−^) production could be converted in H_2_O_2_, we measured mitochondrial O_2_^−^ using the mitochondria-specific probe MitoSOX Red^23^. After treatment with H_2_O_2_ or rotenone/Ant^24^, MitoSOX-specific fluorescence was increased more markedly in CCR6^+^Th17 cells compared to CCR6^neg^Th cells, suggesting a higher mitochondrial basal activity in CCR6^+^Th17 cells (Fig. 6B). Mitochondrial O_2_^−^ production was not altered in CSE-exposed CCR6^+^Th17 and CCR6^neg^Th cells; CSE exposure decreased MitoSOX-specific fluorescence in rotenone/Ant- pretreated CCR6^+^Th17 and CCR6^neg^Th cells, and abolished the difference in MitoSOX-specific fluorescence intensity observed between these two subpopulations. Coherently, the expression of NAD(P)H:quinone oxidoreductase (NQO1) and heme oxygenase-1 (HO-1), two major NF-E2-related factor 2 (Nrf2) regulated anti-oxidant phase II genes^25^, was more abundant in CSE-exposed CCR6^+^Th17 cells than in CCR6^neg^Th cells (Fig. 6C).

## Discussion

Although the literature is rich in defining the immunosuppressive role of cigarette smoke exposure on TCR-reactivated antigen-specific Th cells^3^, its effect on memory resting Th cells in a quiescent state is not known. Through the study of peripheral CCR6^+^ Th17 cells^26^ exposed to cigarette smoke independently of TCR stimulation, we provide evidences that higher sensitivity to CSE-induced premature senescence of resting CCR6^+^ Th17 cells compared to other memory CD4^+^ T cells relies on higher basal ROS production and dependent signalling in these cells. Moreover, CSE-induced premature senescence of CCR6^+^Th17 is associated with VEGFα production, which could confer pathogenicity to CCR6^+^Th17 cells and contribute to worsen chronic inflammation in smokers with autoimmune diseases when homing to mucosal tissues.

Increased number and ratio of CCR6^+^Th17 cells have been found in healthy smokers and patients with COPD, and associated with the capacity of dendritic cells of smokers to bias differentiation of naïve CD4^+^ T cells toward a Th17 phenotype^27^. Considering elevated levels of senescent pulmonary cells in smokers with COPD^9–11,28^, our current findings that acute CSE exposure induces CCR6^+^Th17 cells premature senescence can at least partially also explain the resultant systemic accumulation of CCR6^+^Th17 cells^4–7^, and contribute to higher CCR6^+^/CCR6^−^ T cells ratio in healthy smokers. Our data are in accordance with other reports showing that accumulation of p16^INK4a^ - expressing T cells in human peripheral blood is a biomarker of physiologic age as opposed to chronologic age, and independently correlates with gerontogenic behaviors including smoking^29,30^. The composition of the secretome of senescent cells depends on the stimuli triggering senescence, and is also specific of cell type^12^. This could explain the restricted secretory phenotype of isolated CCR6^+^Th17 cells exposed to CSE, consisting of combined VEGFα and IL-8 expression, contrasting with FoxP3^+^ regulatory T cells induced- senescent CD4^+^ T cells which produce other classical SASP factors such as TNFα, Il-1β and IL-6^17^.

We further demonstrated that higher sensitivity of CCR6^+^Th17 cells to premature senescence relied on a higher basal production of ROS resulting in ROS dependent -senescence after CSE exposure, as already shown for various cell types^28^. Higher basal production of ROS could contribute to the TCR-independent higher tonic ERK1/2 signalling in resting CCR6^+^ Th17^18^, leading to p16^INK4a^ expression after ERK1/2 activity reinforcement by CSE-induced ROS overproduction^31^.

By using different pharmacological inhibitors, our results suggest a dichotomic regulation of expression of p16^INK4a^, a growth arrest-related characteristics of senescence, and VEGFα production by CSE-induced senescent CCR6^+^Th17 cells, as already reported for the SASP of other cell types^32,33^. In support of this is the selective inhibition of CSE-induced production of VEGFα by NAC pretreatment, in comparison with the antimycin A -sensitivity of CSE-stimulated p16^INK4a^ expression. Of note, CSE-modulation of antimycin A-sensitivity in CCR6^+^Th17 cells was not associated with enhancement of mitochondrial superoxide formation as already shown in epithelial cells^34^ suggesting that basal respiratory chain activity of CCR6^+^Th17 cells was not sensitive to CSE. In contrast, antimycin A+ rotenone treatment increased mitochondrial superoxide formation in CCR6^+^Th17 cells, and this mitochondrial activity was suppressed by CSE exposure, suggesting that CSE induced dysfunction of respiratory chain activity in CCR6^+^Th17 cells, only when respiratory chain was activated beforehand. Moreover, our results suggest that CSE-induced VEGFα production could result from ROS-activated ERK1/2 signalling cooperating with overactivation of the antioxidant Nrf2 axis^35,36^. Overactivation of the antioxidant Nrf2 axis in CSE-exposed CCR6^+^Th17 cells could also account for the repression of TNFα-, IL-6 & IL-1β expression as recently described in macrophage^37^. In conclusion, the functional characteristics of CSE-exposed CCR6^+^Th17 cells, both inflammatory and angiogenic, could contribute to the pathogenic potential of cigarette smoking in susceptible patients, as described for circulating angiogenic T cells in encephalomyelitis, systemic lupus erythematosus, systemic sclerosis and rheumatoid arthritis^38,39^. Confirming our previous data on radiation-induced senescent CCR6 + Th17 cells^40^, our results also suggest that secretion of VEGFα by mucosa-homing CCR6^+^Th17 cells submitted to any oxidative microenvironments, independently of TCR-reactivation, could represent a potential damage response altering insulted and nearby tissue. This damage response should be addressed independently from cell-cycle regulation or senescence *per se*, for discovering new targets controlling senescence effector response in diverse chronic inflammatory disorders both environmental- and age-related.

## Methods

### Isolation of CD4^+^ T lymphocyte subsets

Peripheral blood mononuclear cells (PBMCs) of healthy donors from the French Blood Bank (Etablissement Français du Sang, Creteil, France) were separated using UNI-SEP Separation Tubes (Novamed, Jerusalem, Israel), and CD4^+^ T cells were further enriched by negative selection using a CD4^+^ T cell isolation kit (Miltenyi Biotec, Bergisch Gladbach, Germany). CD4^+^ CD45RO^+^ CD127^−^ CD25^high^ Tregs, CD4^+^ CD45RO^+^ CD127^+^ CD25^low^ CCR6^+^ Th17 cells and CD4^+^ CD45RO^+^ CD127^+^ CD25^low^ CCR6^negative^Th cells were highly purified (purity ≥99%) according to the cell sorting strategy indicated in Fig. S2A, from the CD4^+^ T cells enriched fraction stained with monoclonal antibodies (mAbs) against CD127 PE (R34.34), CD45RO ECD (UCHL1, Beckman Coulter, Villepinte, France), CD196 (CCR6) BV 421 or PE-Cy7 (11A9), CD25 APC mAbs (2A3, BD Biosciences France, Le Pont de Claix, France), CD45RO FITC (UCHL1, Miltenyi Biotec), and CD4 APC eFluor 780 (RPA-T4, eBiosciences, San Diego, CA USA), using a fluorescence-activated cell sorter (Influx; BD Biosciences). After sorting, cells were plated at 10^6^ cells /ml in 96-well U bottom plates (Corning, Acton, MA USA) and put at 37 °C to rest overnight in complete medium within 5% CO2 incubator. Viability and proliferation of cells were analyzed by enumeration in a haemocytometer of Trypan blue-diluted cells and 7-AAD labeling **(**Fig. S1**)**. We added 7-AAD to a final concentration of 0.5 µg/ml just prior to acquisition to exclude dead cells from flow cytometric analysis. RPMI 1640 Medium GlutaMAX™ Supplement HEPES 25 mM [Thermo Fisher, Waltham, MA USA], supplemented with 1 mM sodium pyruvate, 1% MEM Non-Essential Amino Acids Solution, 100 U/ml penicillin, 100 μg/ml streptomycin, and 10% human AB serum or 2.5% FBS is referred as “complete medium”.

### Cells treatment with cigarette smoke extract and pharmacologic inhibitors

Cigarette smoke extract (CSE) was generated from research-grade cigarettes (3R4F; University of Kentucky, Lexington, KY USA) by repeating bubbling cycles (30 s duration) of 40 mL puff volumes mainstream smoke from one cigarette into 10 mL of serum free culture medium. The CSE was collected (100% concentration) supplemented with 10% human AB serum or 2.5% FBS, adjusted to pH 7.4 and then passed through a 0.22 μm pore size filter (Millipore Corporation, Billerica, MA, USA). CSE preparations were standardized by measuring the absorbance at 320 nm; only preparations with optical density >0.42 were used. CSE were prepared shortly (<1 h) before being added to cells cultures. Non-toxic doses of CSE were CSE doses with >80% live cells remaining, at the time of experiment: 5% and/or 10% CSE if CSE treatment ≤24 h, 5% CSE for 48 h treatment **(**Fig. S1A**)**. According to figure legends, cells were pretreated (or not) with 10 mM N-Acetyl-L-cysteine (NAC, Sigma Aldrich) for 1h30, 5 μM U0126 (Merck Millipore), 0.5 µM antimycin A or 2 µM rotenone (Sigma Aldrich) for 45 min before CSE exposure.

### RNA isolation and real time quantitative RT-PCR

Total mRNA isolation and qRT-PCR analysis were performed, as previously published^41^. The expression of the indicated target transcripts was measured by the relative quantification of real-time PCR using a mix of each cDNA sample as a calibrator sample, according to the ΔΔCt method^42^.

### Staining for Senescence-associated β-Galactosidase (SA β-Gal), p16^INK4a^, and phospho ERK1/2

Cells were spread on Superfrost plus slides (Menzel-Glaser, Braunshweig, Germany) at 1 × 10^5^ cells/slide. Staining for SA β-Gal activity (Ozyme, Saint-Quentin-en-Yvelines, France) was performed as described^14^. For p16^INK4a^ expression analysis, cells were fixed and permeabilized by treatment, with PBS containing 4% formaldehyde, for 15 minutes at room temperature. The slides were then incubated overnight at 4 °C with anti-p16^INK4a^ (1:250, Abcam, Cambridge, UK) in PBS-Triton X-100 (0,3%) and BSA (0,1%), and subsequently for 1 h at room temperature with Alexa 594 conjugated goat anti-rabbit antibody (1:200, Life Technologies, Saint Aubin, France). Staining for anti-phospho ERK1/2 was performed as described^18^. After washings, slides were mounted with Prolong Gold + DAPI (Life Technologies, Saint Aubin, France) and analyzed on an Axioimager M2 microscope (Carl Zeiss, Oberkochen, Germany).

### Cytokine secretion assays

Culture supernatants were analyzed by Luminex assay (PROCARTAPLEX, Thermo Fisher Scientific, Waltham, MA USA) for the analytes listed in Fig. S1B according to the manufacturer’s instructions.

### Detection of intracellular reactive oxygen species

Intracellular ROS levels were assessed by analyzing oxidation of H_2_DCF-DA (2′7′-dichlorofluorescin diacetate diacetate, Thermo Fisher Scientific), probed in enriched CD4^+^ T cells from PBMCs. Cells were incubated with 0.25 μM H_2_DCF-DA for 45 min at 4 °C in the dark, then exposed or not to CSE in the presence of 0.25 μM H_2_DCF-DA for 30 min. 250 µM *tert*-butyl H_2_O_2_ (TBHP, Sigma Aldrich) treatment was used as a positive control. Finally, cells were stained with the fluorochrome conjugated mAbs directed against specific cell surface markers and DCF. Mean fluorescence intensity (MFI) was measured in the different CD4^+^ T cells subpopulations by flow cytometric analysis using Cyan ADP LX7 and Summit software (Beckman Coulter). Mitochondrial ROS production was measured by analyzing MitoSOX Red (Thermo Fisher) fluorescence. CCR6 + Th17 and CCR6^negative^ Th sorted cells were exposed or not to CSE at 10^6^ cells/ml, in sealed tubes containing PBS Ca^++^, Mg^++^ and BSA (0.5%), for 3 h. MitoSOX Red, at the final concentration of 2.5 µM, was added to cells for the last 30 min of the culture. MitoSox MFI was acquired on LSR Fortessa X20 and Diva software (BD Biosciences). Statistical analysis of flow cytometry data were performed with FlowJo software (Tree Star, San Carlos, CA, USA). DCF/MitoSox MFI of the different CD4^+^ T cells subpopulations in the different experimental conditions are expressed as percentage of DCF/MitoSox MFI of total CD4^+^ T cells in unstimulated condition.

### Statistical analysis

The sample sizes were dependent on the experimental question and are shown in the related figures. 32 individual healthy donors were studied for all experiments shown in this manuscript. At least, 4 individual healthy donors were used for the study of each marker of senescence. Each donor was used to test two different biomarkers at least, depending on the yield of T cells sorting. Statistical analysis tests were performed using Prism version 5.04 (GraphPad, La Jolla, CA, USA) and data are represented as mean standard error of the mean (SEM). As the gaussian distribution of the different biomarkers could not be checked, non-parametric tests were used to address their statistical relevance. The Mann-Whitney test was used to compare the means of two groups of ordinal (non-parametric) data, and the Kruskal-Wallis test (non-parametric test) was used to compare between the 3 groups.

### Ethics approval and consent to participate

Buffy coats from healthy donors were obtained from Etablissement Français du Sang (Créteil, France) in accordance with Institut National de la Santé et de la Recherche Médicale ethical guidelines. According to French Public Health Law (art L 1121-1-1, art L1121-1-2), written consent and Institutional Review Board approval are not required for human non-interventional studies.

## Supplementary information

Supplementary information.
