# Supplementary material for: Cigarette smoking induces human CCR6+Th17 lymphocytes senescence and VEGF-A secretion
Source: Sci Rep. 2020 Apr 16;10:6488. doi: 10.1038/s41598-020-63613-4 (PMC7162978; doi:10.1038/s41598-020-63613-4)
Supplement: Supplementary file 1 — Supplementary information. [file 41598_2020_63613_MOESM1_ESM.pdf]

"Cigarette smoking induces human CCR6$^+$Th17 lymphocytes senescence and VEGF-A secretion".

Indoumady Baskara,[1,2*] Stéphane Kerbrat,[1,2*] Maylis Dagouassat,[1,2] Hoang Quy Nguyen,[1,3] Maude Guillot-Delost,[4,5] Mathieu Surenaud,[6] Claude Baillou,[7] François M. Lemoine,[7] Didier Morin,[1,8] Jorge Boczkowski,[1,2] and Sabine Le Gouvello[1,2,9]

A

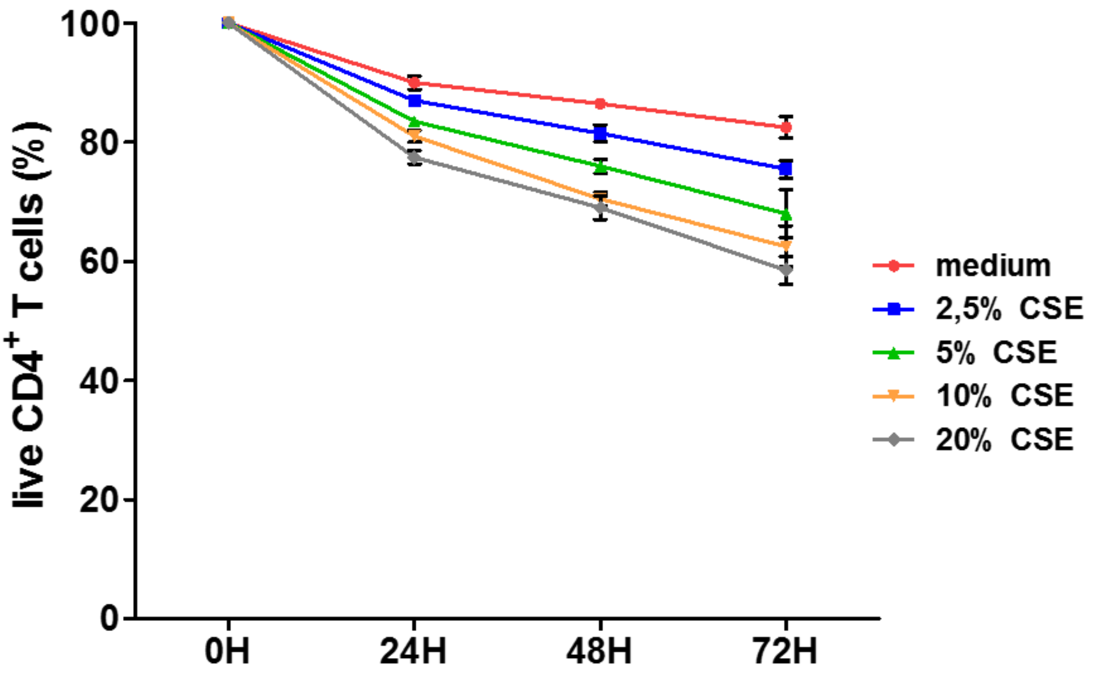

B

Interleukins (IL)	Chemokines (CXCL, CCL)	Other inflammatory factors	Growth factors
IL-1α	IL-8 (CXCL8)	IFNγ	FGF
IL-1β	MCP-1 (CCL2)	LTα	G-CSF
IL-2	MIG (CXCL9)		GM-CSF
IL-4	MIP-1α (CCL3)		TGF-β1
IL-5	MIP-1β (CCL4)		VEGF-α
IL-6	MIP-3α (CCL20)		
IL-7	RANTES (CCL5)		
IL-9			
IL-10			
IL-13			
IL-17A			
IL-21			
IL-22			
IL-23p40			

C

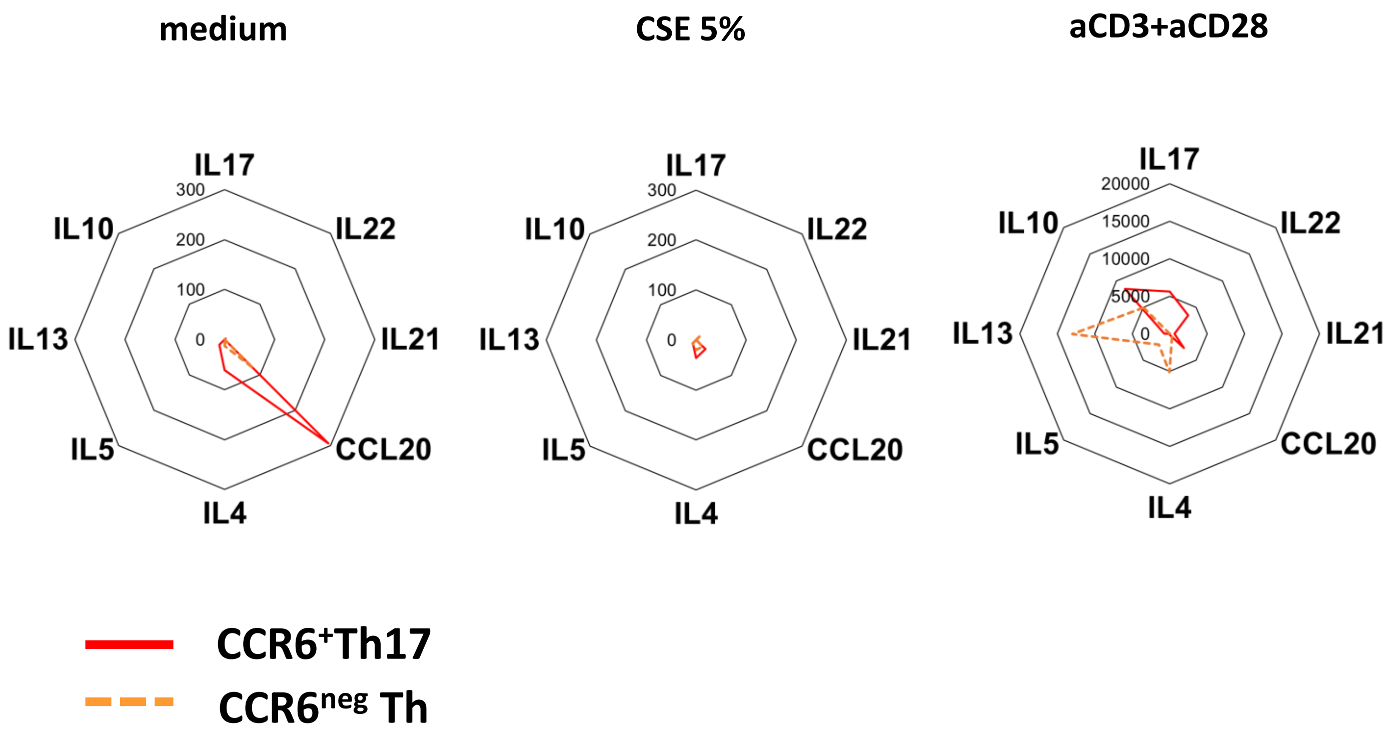

Figure S1

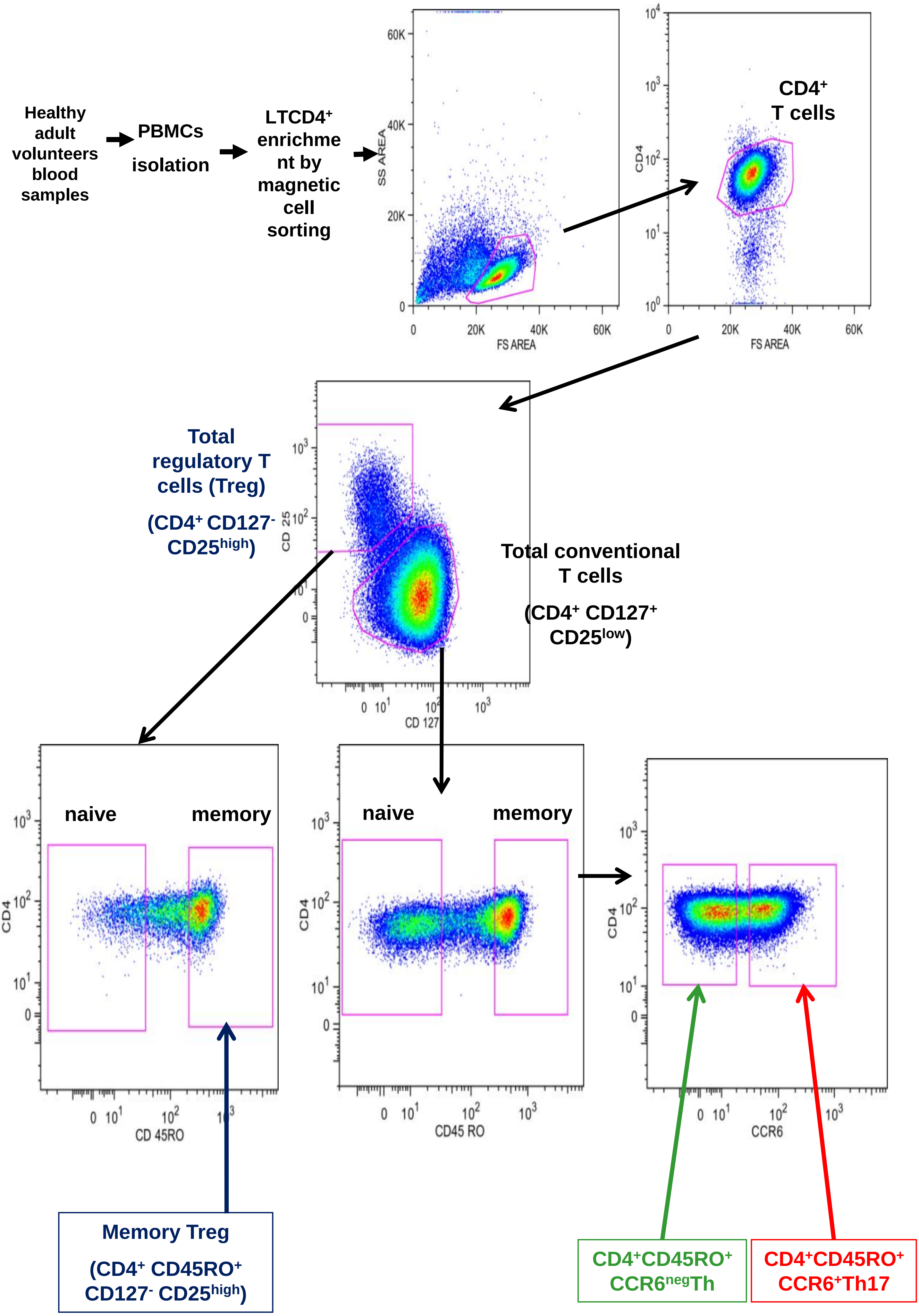
Healthy adult volunteers blood samples
PBMCs isolation
LTCD4+ enrichment by magnetic cell sorting
SS AREA
FS AREA
CD4
CD4+ T cells
Total regulatory T cells (Treg)
(CD4+ CD127- CD25high)
CD 25
CD 127
Total conventional T cells
(CD4+ CD127+ CD25low)
naive
memory
CD 45RO
CD45 RO
CCR6
Memory Treg
(CD4+ CD45RO+ CD127- CD25high)
CD4+CD45RO+ CCR6negTh
CD4+CD45RO+ CCR6+Th17

Figure S2

Supplementary Figures Legends

Figure S1. Cigarette smoke exposure does not stimulate secretion of Th signature cytokines. (A) Cell viability among CD4+ T cells analyzed at indicated time after exposure to different doses of cigarette smoke extract, by 7- AAD labeling excluding dead cells (n=8). (B) List of different electrolytes quantified by Luminex assay. (C) Cigarette smoke exposure does not stimulate secretion of Th signature cytokines. Secretion of Th signature cytokines was quantified by Luminex assay in supernatants of cells cultured in indicated conditions for 48h (n=8).

Figure S2. Gating strategy for sorting of Tregs, memory $CCR6^{+}$ conventional Th17 cells and memory $CCR6^{neg}$ conventional Th cells
